# Trends in HIV Incidence, Prevalence, Antiretroviral Therapy Coverage and Mother-to-Child Transmission Among Pregnant and Breastfeeding Women in the Eastern Cape Province, South Africa, 2000–2023

**DOI:** 10.3390/tropicalmed11070198

**Published:** 2026-07-15

**Authors:** Tronic Sithole, Ziphelele Peter

**Affiliations:** 1 Faculty of Medicine and Health Sciences, School of Public Health, Walter Sisulu University, Mthatha 5100, South Africa; zpeter@wsu.ac.za; 2 Global Centre for Human Resources for Health Intelligence, Walter Sisulu University, East London 5247, South Africa; 3 WSU Society and Health Research Institute, Walter Sisulu University, Mthatha 5117, South Africa

**Keywords:** HIV incidence, pregnant women, breastfeeding, Eastern Cape, mother-to-child transmission, antiretroviral therapy

## Abstract

Background: The Eastern Cape Province carries the second-highest antenatal HIV prevalence nationally (32.9%), yet province-level longitudinal data on HIV incidence among pregnant and breastfeeding women (PBW) remain limited. This study examined trends in HIV incidence, HIV prevalence, mother-to-child transmission (MTCT), and antiretroviral therapy (ART) coverage among PBW in the Eastern Cape from 2000 to 2023. Methods: A quantitative ecological design was employed using secondary analysis of modelled estimates from the Thembisa Provincial HIV Model, version 4.8. Annual HIV incidence in PBW, HIV prevalence in pregnant women (overall and by five-year maternal age group), MTCT rate, new MTCT cases, and ART coverage were extracted with 95% confidence intervals (CIs) for the Eastern Cape, 2000–2023. Descriptive analysis characterised temporal trends at seven reference years, and formal trend significance was assessed using the Mann–Kendall test and log-linear regression. Results: HIV incidence in PBW declined from 3.8% (95% CI: 3.7–3.9) in 2000 to 1.8% (95% CI: 1.3–2.4) in 2023, a relative reduction of approximately 53%. ART coverage rose from 0% to 71.6%, coinciding with an 88% reduction in MTCT rate from 31.3% to 3.6%. By 2023, the MTCT rate had crossed below the World Health Organization 5% elimination threshold. HIV prevalence among pregnant women remained high at 25.0% despite declining incidence. The age distribution of HIV burden shifted markedly toward older maternal cohorts: prevalence among women aged 40–49 years increased from 9.9% in 2000 to 46.5% in 2023, while prevalence in the 15–24 age group declined substantially. New MTCT cases fell from 9990 in 2000 to 1236 in 2023. Conclusions: Declining HIV incidence in PBW coincided with substantial ART scale-up in the Eastern Cape, while HIV prevalence among pregnant women remained persistently high, a divergence that reflects the accumulating, ART-sustained pool of women living with HIV who survive into older reproductive age rather than a reversal of programmatic progress; this divergence between declining incidence and persistent, ageing prevalence is the central epidemiological finding of this study. Targeted interventions, including age-responsive antenatal protocols and strengthened PMTCT retention, alongside more careful consideration of expanded pre-exposure prophylaxis (PrEP) access, are needed to achieve elimination of mother-to-child transmission.

## 1. Introduction

South Africa continues to experience one of the largest HIV epidemics globally, accounting for a disproportionate share of new infections and people living with HIV in sub-Saharan Africa. While national prevention and treatment programmes have produced measurable gains in epidemic control, substantial disparities persist across population groups and geographic regions [1,2]. The Eastern Cape Province carries a particularly high antenatal HIV burden, with the 2022 National Antenatal HIV Sentinel Survey estimating an antenatal HIV prevalence of 32.9%, the second highest of all nine provinces and well above the national average of 27.5% [3]. These rates exceed HIV prevalence among adult women (22.3%) and adults aged 15–49 years (17.0%) in the general population, placing pregnant women at the centre of the province’s HIV response [4]. This persisting prenatal burden underlines the need for preventative efforts that go beyond clinical service delivery to address the social and structural factors that influence women’s HIV risk and engagement with care.

Pregnant and breastfeeding women (PBW) face elevated HIV acquisition risk through a combination of biological, behavioural, and structural pathways [5,6]. Pregnancy-related hormonal changes alter vaginal microbiome composition and mucosal immunity in ways that may increase susceptibility to HIV, while partner behaviour during pregnancy and extended breastfeeding maintains sustained transmission risk [7]. A systematic review and meta-analysis of 37 studies across sub-Saharan Africa estimated an average HIV incidence among PBW of 3.6 per 100 person-years (95% prediction interval: 1.2–11.1), exceeding the World Health Organization’s threshold for substantial HIV acquisition risk [7]. Average incidence was higher in studies conducted before 2010 (4.1/100 person-years) and lower in the post-2014 period (2.1/100 person-years), reflecting the downstream benefits of combination HIV prevention on maternal populations [7]. These patterns underline the importance of comprehensive health promotion approaches that integrate biomedical prevention with gender-responsive counselling, partner engagement, and community-based risk reduction throughout pregnancy and breastfeeding.

South Africa’s Prevention of Mother-to-Child Transmission (PMTCT) programme has evolved, progressing from regimen-based prophylaxis to universal Option B+ from 2015 and the ‘treat all’ policy from 2016 [8,9]. Evidence from 41 sub-Saharan African countries demonstrates that ART coverage during pregnancy is a primary determinant of MTCT at the population level, with each 1% increase in antenatal ART coverage associated with a 0.18% reduction in MTCT rate (95% CI: −0.19 to −0.16, *p* < 0.001) [10]. Despite these programme gains, national progress toward the UNAIDS 95-95-95 targets among pregnant women remains incomplete, with only two-thirds of pregnant women living with HIV achieving viral suppression [4,11]. Evidence from north-eastern South Africa further shows that almost half of HIV-positive pregnant women do not maintain optimal ART coverage across the full vertical transmission risk period, due to treatment interruptions, late initiation, and loss to follow-up [12].

Province-level longitudinal analyses of HIV incidence trends specifically among PBW in the Eastern Cape remain scarce. The Thembisa Provincial HIV Model provides calibrated annual estimates at the provincial level for a comprehensive range of HIV epidemiological and programme indicators, offering a unique resource for tracking epidemic trajectory in this high-burden setting [13]. This study used Thembisa version 4.8 to examine trends in HIV incidence, HIV prevalence, MTCT rates, and ART coverage among PBW in the Eastern Cape from 2000 to 2023. The central research question was: did HIV incidence among PBW in the Eastern Cape decline between 2000 and 2023, and what was the relationship between ART coverage and MTCT outcomes over this period? The objectives were to: (1) assess temporal changes in HIV incidence and prevalence among PBW; (2) examine trends in MTCT rate and absolute case burden; (3) characterise the shifting age distribution of HIV prevalence among pregnant women; and (4) describe the relationship between ART coverage expansion and key maternal HIV outcomes.

## 2. Materials and Methods

### 2.1. Study Design

A quantitative ecological design was employed using secondary analysis of nationally modelled HIV epidemiological estimates from the Thembisa Provincial HIV Model, version 4.8. The analysis examined temporal trends in HIV incidence and related maternal HIV outcomes among PBW in the Eastern Cape Province over 24 years from 2000 to 2023. An ecological design was appropriate because the study used aggregated population-level model estimates rather than individual-level data. In this design, the unit of analysis is the population (the Eastern Cape Province as a whole) observed at successive time points, rather than individual women followed over time; exposure (e.g., ART coverage) and outcome (e.g., HIV incidence) are both measured as aggregate annual estimates and compared across time rather than linked at the level of a single person. This approach is well-suited to characterising population-level trends and the temporal co-occurrence of programme scale-up with epidemiological change, but it cannot establish individual-level causal pathways and is subject to the ecological fallacy, whereby associations observed at the population level may not hold, or may differ in direction or magnitude, at the individual level. This limitation is revisited in the Limitations section.

### 2.2. Data Source

HIV epidemiological estimates were obtained from the Thembisa model (version 4.8), developed by [13] at the University of Cape Town. Thembisa is a nationally validated mathematical model used to generate HIV epidemiological indicators for South Africa, calibrated to observed national and provincial surveillance data, including antenatal clinic surveys, population-based surveys, demographic records, mortality data, and national HIV programme statistics. The model generates annual HIV epidemiological estimates for the general population and key population sub-groups, with corresponding 95% CIs. Provincial-level estimates are provided separately for each of South Africa’s nine provinces. The 95% CIs in this study are model-derived parametric uncertainty ranges reflecting uncertainty within the Thembisa framework, not empirical sampling variance. All estimates used in this study are retrospective model fits to observed data through 2023.

### 2.3. Study Population

The study population comprised PBW in the Eastern Cape Province. For comparative purposes, HIV prevalence among pregnant women was also extracted stratified by five-year maternal age group (15–19, 20–24, 25–29, 30–34, 35–39, and 40–49 years). Overall, ART coverage for the Eastern Cape province was extracted as the primary measure of programme scale-up.

### 2.4. Study Variables

The primary outcome was HIV incidence in PBW, expressed as a proportion per year with 95% CI. Additional variables extracted included: HIV prevalence in pregnant women (mean and 95% CI), overall and by maternal age group; MTCT rate (mean and 95% CI), defined in the model as the proportion of children newly infected through vertical transmission per HIV-positive woman delivering in the previous 12 months; overall ART coverage (mean and 95% CI); new MTCT cases (mean); total births to HIV-positive mothers (mean); and new HIV infections attributed to breastfeeding versus at or before birth (mean), providing a decomposition of vertical transmission route.

### 2.5. Data Analysis

Descriptive statistical analysis was conducted to examine trends in each indicator over the study period. Absolute changes were calculated by subtracting estimates at each reference year from the 2000 baseline. Relative changes were expressed as percentage reductions from baseline. Seven reference years (2000, 2005, 2010, 2015, 2019, 2022, and 2023) were selected for tabular presentation, capturing the pre-ART era, early rollout, programme expansion under Option B, Option B+ implementation, and the most recent retrospective estimates. Age-disaggregated HIV prevalence data were presented at the same reference years to characterise the shifting maternal age distribution of HIV burden. All data management and analysis were conducted in Microsoft Excel and Stata version 19.

To formally quantify the temporal trends observed in HIV incidence, MTCT rate, and ART coverage, the full annual series (2000–2023) was further analysed using the non-parametric Mann–Kendall trend test, which assesses the presence and direction of a monotonic trend without assuming a particular functional form, together with Sen’s slope estimator. The average annual percentage change (AAPC) for each indicator was additionally estimated using log-linear regression of the indicator on calendar year, with AAPC calculated as (exp(β) − 1) × 100, where β is the regression slope; 95% confidence intervals for AAPC were derived from the standard error of β. To explore the temporal relationship between ART coverage and the other two indicators, Pearson correlation coefficients were calculated between annual ART coverage and annual PBW incidence, and between annual ART coverage and annual MTCT rate. Because all three series are smooth, monotonically trending model outputs rather than independent empirical observations, these correlations were interpreted with caution as descriptive measures of co-movement over time rather than as evidence of a causal or statistically independent association; this limitation is revisited in the Results and Limitations sections. Trend and correlation analyses were performed in R version 4.5 (R Foundation for Statistical Computing, Vienna, Austria) using the ‘pymannkendall’ and ‘scipy’ routines.

### 2.6. Justification for the Study Period

The period 2000 to 2023 was selected to capture the full arc of South Africa’s HIV epidemic among PBW, from the pre-ART era through universal treatment. The year 2000 precedes meaningful ART access in the Eastern Cape, providing a robust pre-intervention baseline. The end of 2023 represents the most recent year for which retrospective modelled fits are available from Thembisa version 4.8, before the model transitions to forward projections. This 24-year window enables assessment of the long-term epidemiological impact of the full PMTCT programme evolution.

## 3. Results

**Figure 1:**
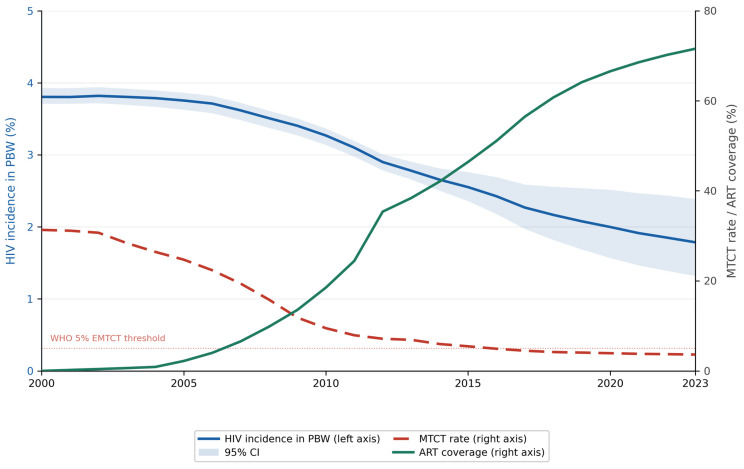
Trends in HIV incidence among pregnant and breastfeeding women (PBW), mother-to-child transmission (MTCT) rate, and antiretroviral therapy (ART) coverage, Eastern Cape Province, 2000–2023. Note. Shaded band = 95% CI for HIV incidence in PBW (left axis, 0–5%). MTCT rate and ART coverage are plotted on the right axis (0–80%). The dotted horizontal line indicates the WHO (2017) 5% EMTCT threshold for breastfeeding populations. Source: Thembisa Provincial HIV Model, version 4.8.

This study analysed HIV incidence, prevalence, and MTCT trends among PBW in the Eastern Cape Province from 2000 to 2023. Annual estimates for key indicators at seven reference years are presented in Table 1. Figure 1 displays simultaneous trends in PBW incidence (with 95% CI shading), MTCT rate, and ART coverage over the full study period. Age-disaggregated HIV prevalence among pregnant women is presented in Table 2.

HIV incidence in PBW in the Eastern Cape reached its peak at approximately 3.8% per year (95% CI: 3.7–3.9) in 2000 and declined progressively thereafter (see Table 1). The rate of decline was gradual between 2000 and 2009, then accelerated as ART coverage expanded from 2010 onward. By 2023, incidence had fallen to 1.8% (95% CI: 1.3–2.4), a relative reduction of approximately 53%. A notable feature of the post-2015 estimates is the progressive widening of the 95% CI, indicating increased model uncertainty in the more recent calibration period (see Figure 1). The annual incidence rate remained above the WHO substantial risk threshold of 1.0 per 100 person-years throughout the study period.

HIV prevalence in pregnant women rose from 20.0% (95% CI: 19.2–20.7) in 2000 to a plateau of 26.7–26.8% between 2007 and 2017, before declining modestly to 25.0% (95% CI: 22.8–27.6) by 2023 (see Table 1). Unlike incidence, which declined, prevalence remained persistently elevated throughout the ART era: one in four pregnant women in the Eastern Cape was HIV-positive in 2023.

The MTCT rate fell from 31.3% (95% CI: 30.5–32.1) in 2000 to 3.6% (95% CI: 3.2–4.2) in 2023, an 88% reduction (see Table 1; Figure 1). This decline tracked closely with ART coverage, which rose from 0% in 2000 to 71.6% (95% CI: 69.1–73.8) by 2023. The sharpest absolute reductions in MTCT rate were recorded between 2005 and 2015. By 2023, the Eastern Cape’s MTCT rate had crossed below the WHO (2017) 5% elimination threshold for breastfeeding populations. New MTCT cases peaked at 11,489 in 2005 before declining to 1,236 by 2023, a relative reduction of approximately 89.2%. The 2005 peak exceeded the 2000 figure of 9990, reflecting continued growth in the absolute number of HIV-positive pregnant women during the late pre-ART era, even as the transmission rate was already falling. From 2010 onward, both the rate and absolute number of new MTCT cases declined in parallel.

Formal trend analysis confirmed that all three indicators followed statistically significant monotonic trends across 2000–2023. The Mann–Kendall test indicated a significant decreasing trend in PBW incidence (Kendall’s τ = −0.97, *p* < 0.001; Sen’s slope = −0.108 percentage points/year), a significant decreasing trend in MTCT rate (τ = −1.00, *p* < 0.001; Sen’s slope = −1.355 percentage points/year), and a significant increasing trend in ART coverage (τ = 1.00, *p* < 0.001; Sen’s slope = 3.801 percentage points/year). Log-linear regression yielded an average annual percentage change (AAPC) of −3.67%/year (95% CI: −4.02 to −3.31) for PBW incidence, −10.72%/year (95% CI: −11.69 to −9.74) for MTCT rate, and +28.72%/year (95% CI: 22.57 to 35.18) for ART coverage. Pearson correlation coefficients between annual ART coverage and the other two indicators were r = −0.999 (*p* < 0.001) for PBW incidence and r = −0.893 (*p* < 0.001) for MTCT rate. The near-perfect correlation between ART coverage and PBW incidence should be interpreted cautiously: because both series are smooth, monotonically trending outputs of the same underlying Thembisa model rather than independent empirical measurements, this coefficient quantifies the degree to which the two series co-trend over the study period and is expected to be high by construction; it does not, on its own, constitute evidence of a causal or even a statistically independent association between ART coverage and incidence decline (see Limitations).

The age distribution of HIV burden among pregnant women changed markedly over the 23 years (see Table 2). In 2000, the highest burden was concentrated among women aged 20–24 years (27.8%), with prevalence declining across older age groups. By 2010, the distribution had shifted, with women aged 30–34 and 35–39 years now recording the highest prevalences at 36.6% and 30.6%, respectively. By 2023, the burden had shifted further toward older reproductive cohorts: women aged 35–39 and 40–49 years recorded the highest prevalences at 42.8% and 46.5%, respectively, while women aged 15–19 and 20–24 years declined to 10.4% and 18.8%, respectively.

## 4. Discussion

This study examined trends in HIV incidence, prevalence, and MTCT among PBW in the Eastern Cape Province from 2000 to 2023. HIV incidence declined by approximately 53% over the period, while the MTCT rate fell by 88%, crossing below the WHO [14] 5% elimination threshold by 2023. These findings are consistent with evidence from sub-Saharan Africa demonstrating that ART scale-up coincided with, and is regarded as a primary contributor to, reductions in maternal HIV incidence and vertical transmission at the population level [10,15]; as an ecological, model-based analysis; however, this study cannot itself establish a causal relationship between ART scale-up and the observed declines.

The temporal alignment between ART scale-up and reductions in both PBW incidence and MTCT rate provides strong ecological evidence for the population-level impact of South Africa’s PMTCT and treat-all programme. ART coverage rose from effectively zero in 2000 to 71.6% by 2023. Astawesegn et al. [10] demonstrated across 41 sub-Saharan African countries that each 1% increase in antenatal ART coverage corresponded to a 0.18% reduction in MTCT rate, operating through viral load suppression that reduces viral concentrations in genital secretions and breast milk. Despite the 88% reduction in MTCT rate, over 1200 new child HIV infections occurred in the Eastern Cape in 2023, indicating that elimination remains aspirational. Residual MTCT cases reflect incomplete ART coverage, women newly acquiring HIV during pregnancy or breastfeeding, and postpartum retention gaps. Etoori et al. [12] demonstrated that almost half of HIV-positive pregnant women in north-eastern South Africa did not achieve optimal ART coverage across the full MTCT risk period, with early stable ART reached in only 51.9% of pregnancies.

While new infections in PBW fell by more than half, HIV prevalence among pregnant women remained at approximately 25%, comparable to the 28.6% recorded in the Philani Ndiphile cohort in Buffalo City Metro, Eastern Cape [4], a divergence between declining incidence and persistently high prevalence that constitutes one of the central findings of this analysis. This divergence arises because prevalence reflects the accumulated stock of established infections, sustained by ART extending the survival and fertility of women living with HIV into their late reproductive years, while incidence captures only the annual flow of new acquisitions [3]. Three interrelated mechanisms help explain the specific shape of the prevalence trajectory observed here—rising from 20.0% in 2000 to a plateau of 26.7–26.8% between 2007 and 2017, before declining modestly to 25.0% by 2023. First, ART-related survival gains progressively enlarged the pool of women living with HIV who survive into, and beyond, their reproductive years; as mortality among women on treatment fell, women who would previously have died before or during their reproductive window instead remained alive and at risk of pregnancy, mechanically inflating the prevalent pool even as new infections declined. Second, demographic and fertility dynamics reinforced this effect: as the cohorts of women infected during the high-incidence years of the early 2000s aged into their thirties and forties, many remained fertile and continued to fall pregnant, so that the prevalent pool of pregnant women living with HIV came increasingly to reflect older, ART-treated survivors rather than newly infected younger women (see also the age-stratified findings below). Third, there is an inherent lag between changes in incidence and changes in prevalence: because prevalence is a stock built up from many years of past infections, a given year’s decline in new infections takes years, and in this case more than a decade, to be reflected as a corresponding decline in prevalence, since the existing stock of infections must first be depleted through mortality, ART-related survival extending that depletion further, or ageing out of the reproductive age range. Taken together, these mechanisms indicate that the recent modest decline in prevalence (from 26.8% in 2017 to 25.0% in 2023) most plausibly reflects the cumulative downstream effect of incidence reductions that began more than a decade earlier, rather than a recent acceleration in prevention impact. This distinction is critical for service planning: declining incidence does not rapidly translate into declining PMTCT service demand. The Eastern Cape’s antenatal PMTCT caseload will remain high for at least a decade even if new infections were eliminated tomorrow, given the scale of the existing stock of HIV-positive women of reproductive age.

A further notable pattern is the near-reversal of the age gradient in HIV prevalence among pregnant women over the study period. In 2000, the 20–24 age group carried the highest burden (27.8%); by 2023, women aged 35–49 recorded the highest provincial prevalences (42.8% and 46.5%, respectively). Babalola et al. [4] similarly documented a steep age gradient in the Philani Ndiphile cohort, with prevalence increasing by approximately 4.8 percentage points per additional year of age, attributing this pattern to prolonged sexual activity, accumulated HIV-positive partner exposure, and the ageing of women who acquired HIV during the epidemic peak. Crucially, this age shift also reflects the cumulative effects of structural determinants that compound HIV risk and influence women’s capacity to consistently engage with prevention and treatment services throughout their lives. These determinants include intimate partner violence, chronic poverty, and long-standing disparities in access to timely, continuous healthcare [4,16].

These findings have direct implications for antenatal care delivery. Babalola et al. [4] found that ART coverage improved progressively with age in the Philani cohort, from 60% at age 18 to over 80% at age 30, while Etoori et al. [12] found that younger women were more likely to experience unstable care engagement and postnatal seroconversion. Differentiated antenatal care protocols are therefore warranted: intensive incident testing, immediate PrEP linkage, and imbalance retention support for young HIV-negative women at ongoing risk and optimised viral load monitoring and co-morbidity screening for older women with established, long-standing infection.

Despite the overall decline in PBW incidence, the annual rate of 1.8% in 2023 remains approximately 80% above the WHO’s substantial risk threshold of 1.0 per 100 person-years, which is used to guide PrEP eligibility, indicating that a sizeable minority of PBW in the Eastern Cape may meet criteria for biomedical prevention options. This study did not evaluate PrEP uptake, adherence, or effectiveness directly, and this incidence threshold should be read as indicative of unmet prevention need rather than as a formal assessment of the PrEP programme. Nationally, PrEP coverage among eligible pregnant women was only 6.5% in the 2022 Antenatal Survey [4], suggesting that, where eligibility criteria are met, expanding PrEP access may represent one component, among several, of a broader prevention response; safety data for newer PrEP formulations during pregnancy and lactation also remain limited [4,17]. Separately, and independent of PrEP considerations, infections acquired during breastfeeding accounted for a substantial proportion of MTCT cases throughout the study period, reflecting the prolonged postnatal exposure window and the particularly high transmission risk associated with incident maternal infections during lactation. Extending PMTCT coverage and repeat HIV testing through breastfeeding cessation, therefore, remains essential [7].

The persisting HIV risk among PBW in the Eastern Cape is embedded in structural vulnerabilities that biomedical interventions alone cannot address. Poverty, limited education, intimate partner violence, and inequitable sexual relationships compound exposure to HIV and constrain access to prevention services [4,16]. To address the observed age shift in HIV burden and the PrEP implementation gap, health promotion initiatives must include social protection, gender-transformative interventions, and enhanced primary healthcare systems in addition to ART and PrEP delivery.

Male partner engagement, including HIV testing at antenatal visits and linkage of HIV-positive partners to care, remains an underutilised prevention strategy in both the Philani Ndiphile trial context and in north-eastern South African cohort evidence [4,12]. For the implementation of these integrated, person-centred methods, antenatal care continues to be a crucial platform, especially when digital health tools are used to improve follow-up, partner engagement, and continuity of care [12,18].

### Limitations

Several limitations should be considered when interpreting these findings. First, the analysis relied on modelled epidemiological estimates rather than direct empirical measurement. Although Thembisa integrates multiple national data sources and is widely validated for South African HIV surveillance, estimates for PBW depend on calibration assumptions regarding transmission dynamics, fertility, and service coverage. Second, the widening 95% CIs for incidence estimates from 2015 onward reflect reduced recent calibration data and should temper confidence in the most recent point estimates. Third, the ecological design used aggregated provincial-level data, precluding examination of individual-level risk factors, sub-provincial heterogeneity, or intra-provincial disparities between rural and urban health districts. The Eastern Cape encompasses substantial internal heterogeneity that aggregate estimates cannot capture. Fourth, breastfeeding duration and infant feeding assumptions are embedded in the model and cannot be independently validated from these outputs. Fifth, the temporal associations between ART coverage and incidence do not establish individual-level causality in the absence of experimental design; this applies equally to the descriptive trend statistics and the correlation coefficients reported in the Results, which quantify co-occurrence and co-trending between series over the study period rather than an independently verified, individual-level causal effect of ART on incidence or transmission. The very high correlation between ART coverage and PBW incidence in particular should be read as a property of two smooth, monotonically trending model outputs rather than as proof of a causal mechanism, and all causal language in this manuscript has been framed accordingly. Despite these limitations, provincial modelled estimates provide important insight into long-term trends and programme impact that is unavailable from any other source in this high-burden setting.

## 5. Conclusions

The central epidemiological finding of this study is that HIV incidence among pregnant and breastfeeding women in the Eastern Cape Province declined substantially between 2000 and 2023 alongside progressive ART scale-up, while HIV prevalence among pregnant women remained persistently high and increasingly concentrated among older maternal cohorts, reflecting the growing, ART-sustained pool of women living with HIV who survive into later reproductive years. This divergence between declining incidence and persistent, ageing prevalence, rather than any single policy lever, is the pattern that should anchor service planning going forward. Over the same period, the MTCT rate fell to below the WHO [14] 5% elimination threshold, representing genuine programmatic progress; however, persistently high HIV prevalence among pregnant women, the growing concentration of HIV burden among older maternal cohorts, and continued incident infections well above the PrEP eligibility threshold make clear that elimination of mother-to-child transmission remains unfinished work. These findings carry direct implications for the Eastern Cape Provincial Department of Health’s maternal and child health programming and for South Africa’s National Strategic Plan on HIV, TB, and STIs. At the provincial level, three concrete actions follow from these findings. First, age-responsive antenatal care protocols should prioritise intensive incident-HIV testing and PrEP counselling for women under 30 years, alongside structured viral load monitoring and multimorbidity screening for women over 35 years, who now carry the highest prevalence burden. Second, differentiated PrEP delivery embedded within routine antenatal and postnatal visits, with simplified eligibility screening and same-day initiation where the WHO incidence threshold is met, could help close the gap between the 1.8% PBW incidence observed in 2023 and the 6.5% national PrEP coverage reported among eligible pregnant women. Third, PMTCT retention strategies spanning the full postpartum and breastfeeding period, including structured defaulter tracing and repeat HIV testing, are needed to address the ART retention gaps documented in this and prior Eastern Cape cohorts. To overcome prevention gaps and maintain long-term epidemic benefits, person-centred, socially responsive models bolstered by digital health innovations, community health worker platforms, and improved primary care systems will be crucial. Future research should prioritise primary longitudinal studies with provincial and sub-provincial focus to validate and extend these modelled estimates with directly observed data.

## Figures and Tables

**Table 1:** HIV epidemiological indicators for pregnant and breastfeeding women, Eastern Cape Province, selected years, 2000–2023.

Year	HIV Incidence in PBW, % (95% CI)	HIV Prevalence in Pregnant Women, % (95% CI)	MTCT Rate, % (95% CI)	ART Coverage, %	New MTCT Cases, n
2000	3.8 (3.7–3.9)	20.0 (19.2–20.7)	31.3 (30.5–32.1)	0.0	9990
2005	3.8 (3.6–3.9)	25.4 (24.6–26.3)	24.7 (24.1–25.3)	2.2	11,489
2010	3.3 (3.1–3.4)	26.2 (25.5–27.1)	9.5 (9.1–9.9)	18.5	3430
2015	2.6 (2.4–2.8)	26.7 (25.9–27.6)	5.5 (5.1–5.8)	46.4	2040
2019	2.1 (1.7–2.5)	26.6 (25.3–28.0)	4.1 (3.7–4.6)	64.1	1459
2022	1.8 (1.4–2.4)	25.5 (23.5–27.8)	3.7 (3.3–4.3)	70.2	1289
2023	1.8 (1.3–2.4)	25.0 (22.8–27.6)	3.6 (3.2–4.2)	71.6	1236

**Note.** PBW = pregnant and breastfeeding women; MTCT = mother-to-child transmission; ART = antiretroviral treatment; CI = confidence interval. CIs are model-derived parametric uncertainty ranges. MTCT case counts rounded to the nearest unit. Source: Thembisa Provincial HIV Model, version 4.8.

**Table 2:** Age-specific HIV prevalence (%) among pregnant women, Eastern Cape Province, 2000–2023.

Year	15–19 Years	20–24 Years	25–29 Years	30–34 Years	35–39 Years	40–49 Years
2000	14.8%	27.8%	23.2%	17.1%	13.7%	9.9%
2005	13.1%	29.9%	33.7%	27.5%	21.7%	16.3%
2010	10.2%	24.9%	34.6%	36.6%	30.6%	23.6%
2015	9.9%	20.1%	32.5%	40.9%	41.9%	34.6%
2019	10.7%	18.9%	28.3%	39.1%	45.0%	42.5%
2022	10.5%	18.9%	25.5%	35.5%	43.7%	46.1%
2023	10.4%	18.8%	24.9%	34.0%	42.8%	46.5%

**Note.** All values are modelled estimates expressed as percentages. Source: Thembisa Provincial HIV Model, version 4.8.

## Data Availability

The datasets analysed during the current study are publicly available from the Spotlight HIV Dashboard, which provides national HIV epidemiological estimates derived from the Thembisa model. The data can be accessed through the Spotlight website at https://www.spotlightnsp.co.za/hiv-dashboard/ (accessed on 7 January 2026). The estimates used in this study are available in the public domain and can be accessed without restriction for research and academic purposes.

## Abbreviations

The following abbreviations are used in this manuscript:

HIV	Human Immunodeficiency Virus
MTCT	Mother-To-Child Transmission
PrEP	Pre-Exposure Prophylaxis
PBW	Pregnant and Breastfeeding Women
ART	Antiretroviral Therapy
PMTCT	Prevention of Mother-To-Child Transmission
EMTCT	Elimination of Mother-To-Child Transmission
WHO	World Health Organization
UNAIDS	Joint United Nations Programme on HIV/AIDS
